# Radiographic analysis of the restoration of hip joint center following open reduction and internal fixation of acetabular fractures: a retrospective cohort study

**DOI:** 10.1186/1471-2474-15-277

**Published:** 2014-08-13

**Authors:** Hong-fei Shi, Jin Xiong, Yi-xin Chen, Jun-fei Wang, Yin-he Wang

**Affiliations:** Department of Orthopaedics, Nanjing Drum Tower Hospital, The Affiliated Hospital of Nanjing University Medical School, No. 321 Zhongshan Road, Nanjing, China

**Keywords:** Acetabular fracture, Open reduction and internal fixation, Hip joint center, Radiography

## Abstract

**Background:**

Unfavorable reduction is considered one of the key factors leading to joint degeneration and compromised clinical outcome in acetabular fracture patients. Besides the columns, walls, and superior dome, the postoperative position of hip joint center (HJC), which is reported to affect hip biomechanics, should be considered during the assessment of quality of reduction. We aimed to evaluate the radiographic restoration of HJC in acetabular fractures treated with open reduction and internal fixation.

**Methods:**

Patients with a displaced acetabular fracture that received open reduction and internal fixation in the authors’ institution during the past five years were identified from the trauma database. The horizontal and vertical shifts of HJC were measured in the standard anteroposterior view radiographs taken postoperatively. The radiographic quality of fracture reduction was graded according to Matta’s criteria. The relationships between the shift of HJC and the other variables were evaluated.

**Results:**

Totally 127 patients with 56 elementary and 71 associated-type acetabular fractures were included, wherein the majority showed a medial (89.0%) and proximal (93.7%) shift of HJC postoperatively. An average of 2.8 mm horizontal and 2.2 mm vertical shift of HJC were observed, which correlated significantly with the quality of fracture reduction (*P* < 0.001 for both). The horizontal shift of HJC correlated with the fracture type (*P* = 0.022).

**Conclusions:**

The restoration of HJC correlates with the quality of reduction in acetabular fractures following open reduction and internal fixation. Further studies are required to address the effects of HJC shift on the biomechanical changes and clinical outcomes of hip joint, especially in poorly reduced acetabular fractures.

**Electronic supplementary material:**

The online version of this article (doi:10.1186/1471-2474-15-277) contains supplementary material, which is available to authorized users.

## Background

Acetabular fracture remains as a major challenge to orthopaedic surgeons despite of decades of improvement in its operative management. Following well-planned open reduction and internal fixation (ORIF), a good to excellent result can be estimated in a large part of the patients with acetabular fractures. Meanwhile, the complication rate is still high, which leads to poor long-term outcomes in approximately 20% of the patients[1, 2].

Post-traumatic osteoarthritis, usually accompanied with loss of hip motion and increase of pain, has been considered one of the most common complications associated with compromised outcomes in acetabular fractures[3]. It’s generally accepted that biomechanical alterations in hip joint, caused by an unfavorable fracture reduction, play undoubtable roles in the development of arthritis. In previous studies, special emphases were placed to analyze the changes of intraarticular contact characteristics and the loss of stability after acetabular fractures[4, 5].

The hip joint center (HJC), also known as the rotation center of hip joint, is considered crucial for the biomechanical reconstruction of the hip joint during total hip arthroplasty (THA) and revision surgeries[6, 7]. When an acetabular fracture occurs, it’s not rare that the position of HJC will change following the destruction of acetabulum and innominate bone. Since an unfavorable position of HJC was reported to cause increased hip load, compromised soft tissue balancing, and even gait changes[8, 9], it might contribute to the development of post-traumatic arthritis in patients with acetabular fractures as well. Currently, the postoperative assessment of fracture reduction focuses on the residual displacement of columns, walls, and the superior dome[10, 11]. A clearer understanding of the restoration of postoperative HJC in acetabular fractures, which was merely addressed previously, might shed lights on further optimization of the surgical management. In this study, we aimed to quantify the postoperative shift of HJC radiographically, and to evaluate the relationship between the shift of HJC and the quality of fracture reduction following ORIF of acetabular fractures.

## Methods

We retrospectively reviewed the patients with acetabular fractures that were recorded in the trauma database in the authors’ department. The patients were admitted through emergency department or referred from other hospitals. Totally 201 displaced fractures (193 patients) were considered not fitted for Matta’s criteria of nonoperative treatment[12], and then received ORIF between January 2007 and December 2011. Of these reviewed cases, we included those with a full series of standard radiographs, including pre- and postoperative anteroposterior (AP), iliac oblique and obturator oblique Judet views, as well as preoperative computed tomography (CT) scan of the pelvis. Patients with bilateral acetabular fracture, associated fractures of ipsilateral femoral head, fracture of pelvic ring, or those operated on more than two weeks after injury were excluded. The study protocol was approved by the Medical Ethics Committee of Nanjing Drum Tower Hospital (Ref. No. 113217). The study had adhered to the STROBE guidelines for observational studies.

**Figure 1:**
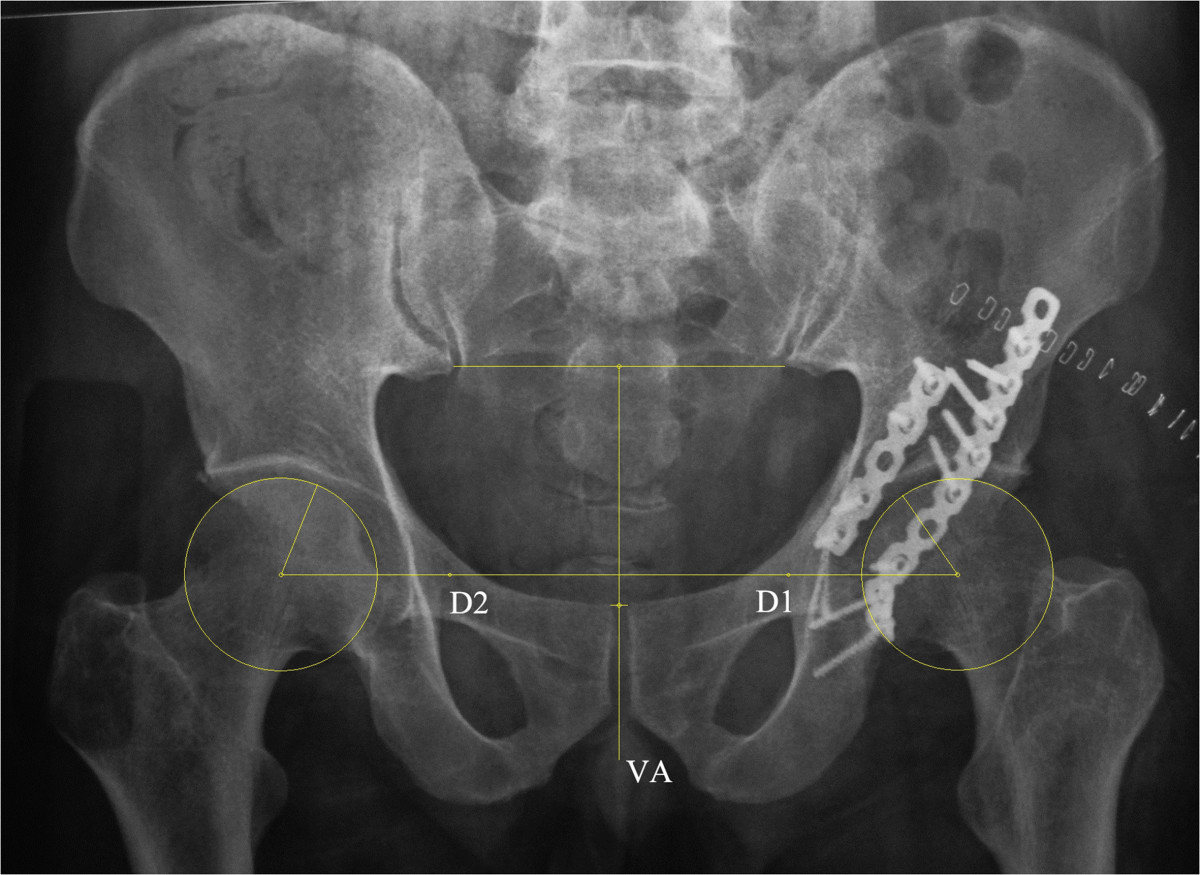
**Radiographic measurement of the postoperative shift of hip joint center (HJC).** The vertical axis of the pelvis (VA line) was defined by connecting the middle of the inter-sacroiliac line and the middle of the pubic symphysis. The distance (D1) between the postoperative femoral head center and the VA line was measured, the same as the distance (D2) between the contralateral intact femoral head center and the VA line. The horizontal shift (X) of the postoperative HJC was calculated as the absolute value of the difference between D1 and D2 (X = |D1 – D2|). The vertical shift (Y) was measured as the distance between the paralleled D1 line and D2 line.

Following thorough preoperative evaluations, all the patients were operated on by two of the senior attendings (JX and YXC). Surgical approaches including Kocher-Langenbeck, ilioinguinal, combined or extensile approaches were determined by the fracture pattern to facilitate reduction and fixation of the innominate bone and the articular surface of acetabulum (Table 1). Definitive fixation was applied with reconstructive plates and screws (Synthes, Switzerland) to stabilize the fracture according to the standard techniques recommended by Letournel[13].Radiographic examination was performed right after the removal of drainage (usually 48 to 72 hours) postoperatively. Standard AP radiograph of the pelvis were taken with the patients placed supine and their feet in a standard position to minimize the effect of rotation of the hip joint. To evaluate the restoration of the HJC following ORIF, we measured the vertical and horizontal shifts of the postoperative center of femoral head from the estimated center of femoral head referring to the contralateral intact hip joint (Figure 1). In brief, the vertical axis of the pelvis (VA line) was defined by connecting the middle of the inter-sacroiliac line and the middle of the pubic symphysis in digitized postoperative AP view radiographs. With built-in tools, the distance (D1) between the postoperative femoral head center and the VA line was measured using Digimizer® image analysis software (MedCalc Software Ltd, Mariakerke, Belgium), the same as the distance (D2) between the contralateral intact femoral head center and the VA line. The horizontal shift (X) of the postoperative HJC was then calculated as the absolute value of the difference between D1 and D2 (X = |D1 – D2|). The vertical shift (Y) was measured as the distance between the paralleled D1 line and D2 line (Figure 1). The direction of the horizontal and vertical shift was also recorded. All the measurements were calibrated with the diameters of the 3.5 mm cortical screws measured in digitized radiographs as reference. Two senior orthopaedic surgeons (JFW and WJW) performed the measurements independently, with the interobserver quantitative data averaged for statistical analysis. The interobserver reliability was examined via interclass correlation coefficient (ICC).

**Table 1:** **Fracture types and information of surgery**

Fracture type	Number	Length of surgery (min)	Surgical approach			
			Ilioinguinal	K-L	Combined	Extensile
Elementary (n = 56)						
Anterior wall	2	245.0 ± 21.2	2			
Anterior column	5	252.0 ± 35.5	5			
Posterior wall	31	106.3 ± 21.2		31		
Posterior column	4	147.5 ± 31.2		4		
Transverse	14	208.9 ± 78.7	3	9	2	
Associated (n = 71)						
Posterior column and posterior wall	3	130.0 ± 8.7		3		
Transverse and posterior wall	15	190.3 ± 87.6		11	4	
T-shape	17	265.6 ± 93.9	2	6	7	2
Anterior column and posterior hemitransverse	12	278.3 ± 62.1	9		3	
Both column	24	338.5 ± 48.4	5		16	3
Summary	127	218.8 ± 102.7^a,b^	26	64	32	5

^a^significant difference among different fracture types (P < 0.05).

^b^significant difference among different surgical approaches (P < 0.05).

The quality of fracture reduction was evaluated by measuring the residual displacement of the columns, walls, and superior dome in digitized anteroposterior (AP) and two oblique (iliac oblique and obturator oblique) Judet view radiographs[12]. The maximum residual displacement (MRD) was used to grade the quality of reduction according to Matta’s criteria: anatomical (MRD less than 2 millimeters), imperfect (MRD between 2 and 3 mm), poor (MRD more than 3 mm), and secondary congruence (articular congruence of the acetabulum whilst displacement of the innominate bone in both-column fractures).

Statistical analysis was performed using IBM SPSS version 19.0 software (SPSS Inc., Chicago, IL), with statistical significance set at a *P* value of less than 0.05. The quantitative data of length of surgery and shift of HJC (X and Y) were demonstrated as mean ± SD and compared among different types of fracture using one-way ANOVA. Person correlation coefficient (*r*) or Spearman’s rank correlation coefficient (rho) was conducted, as appropriate, to identify possible association between the shift of HJC and the other variables. Besides, the relationship of quality of fracture reduction with the other categorical factors was analyzed using Chi-square test. An *a priori* power analysis demonstrated that, with an effect size of 0.3 or greater, a minimum of 84 patients were required to detect a significant correlation between shift of HJC and quality of fracture reduction with 80% statistical power.

## Results

Totally 127 patients (127 fractures) with an average age of 40.2 years (range 17 to 78 years) were included in this investigation, consisted of 82 male and 45 female patients. According to the Letournel and Judet’s classification, there were 56 elementary and 71 associated-type fractures identified in preoperative radiographs and CT images (Table 1). According to our surgical records, the mean length of surgery for all the patients was 218.8 minutes (range 85 to 440 minutes), while significant difference was detected among different fracture types (one-way ANOVA, *P* < 0.001). Comparing the use of different surgical approaches, the length of surgery also varied significantly (one-way ANOVA, *P* < 0.001).

The results of postoperative radiographic evaluation were demonstrated in Table 2. The mean horizontal and vertical shifts (X and Y) of the postoperative HJC were 2.8 mm (range 0.8 to 10.7 mm) and 2.2 mm (range 0.8 to 7.8 mm) respectively, while X showed statistically significant correlation with the fracture type (rho = 0.204, *P* = 0.022). Besides, no correlation was found between the shift of HJC and the surgical approaches. A high interobserver reliability was testified with the ICC of X and Y was 0.88 and 0.81 respectively. Considering the direction of the shift, 113 cases (89.0%) showed a medial shift of postoperative HJC, while 119 (93.7%) of the vertical shift was proximal.

**Table 2:** **Postoperative radiographic evaluation of the shift of hip joint center (HJC) and the quality of fracture reduction**

Fracture type	Postoperative shift of HJC (mm)		Quality of fracture reduction		
	Horizontal	Vertical	Anatomical	Imperfect	Poor
Elementary					
Anterior wall	2.8 ± 1.0	1.9 ± 0.1	2		
Anterior column	2.0 ± 1.3	1.5 ± 0.5	4	1	
Posterior wall	2.0 ± 0.7	1.9 ± 0.7	26	5	
Posterior column	2.4 ± 1.0	2.0 ± 1.2	3	1	
Transverse	3.1 ± 2.3	2.6 ± 1.8	7	5	2
Associated					
Posterior column and posterior wall	1.7 ± 0.5	1.7 ± 0.4	3		
Transverse and posterior wall	2.4 ± 1.4	2.1 ± 1.1	9	5	1
T-shape	3.4 ± 1.8	2.8 ± 2.0	9	6	2
Anterior column and posterior hemitransverse	3.1 ± 2.3	2.0 ± 1.9	6	5	1
Both column	3.6 ± 2.7	2.5 ± 1.5	11	9	4
Summary	2.8 ± 1.9^a,b^	2.2 ± 1.4^b^	80	37	10

^a^significant difference among different fracture types (P < 0.05).

^b^correlation with the quality of fracture reduction (P < 0.05).

**Figure 2:**
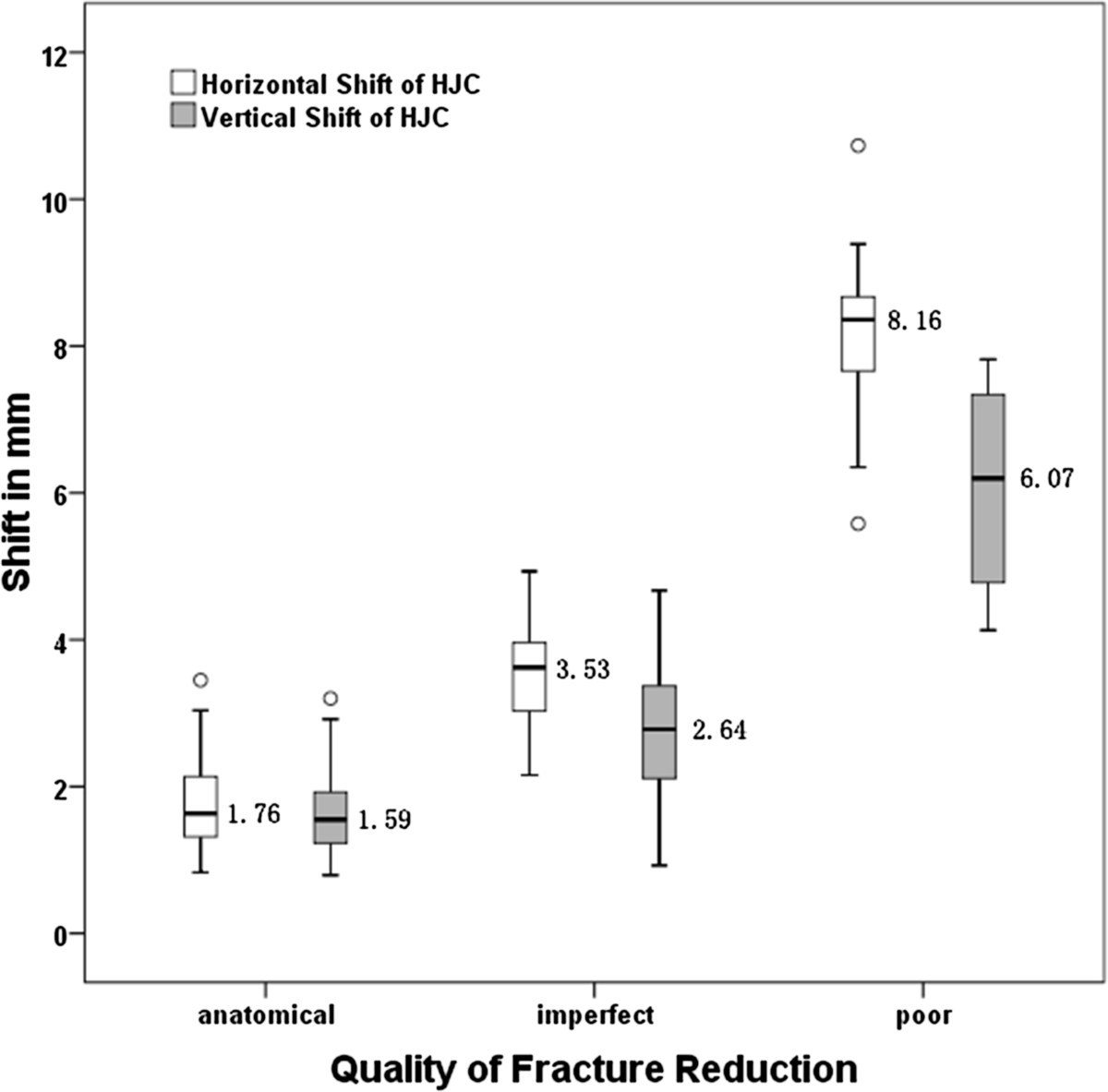
**The horizontal and vertical shift of HJC in patients with different quality of fracture reduction.** The mean values of the shifts were marked beside the boxplot.

**Figure 3:**
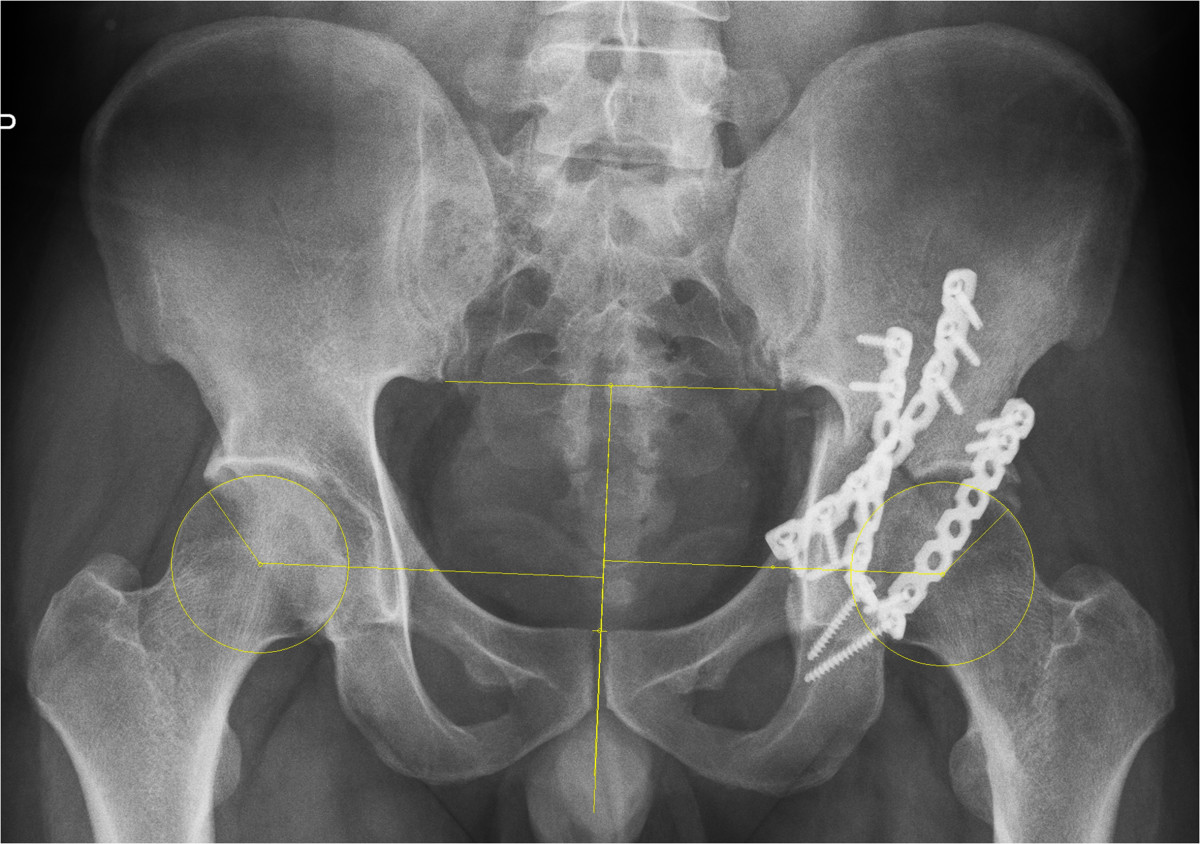
**Postoperative shift of HJC in a poorly reduced acetabular fracture.** The fracture type is transverse and posterior wall. The horizontal and vertical shifts of HJC were measured to be 6.5 mm and 5.8 mm respectively.

The quality of fracture reduction was graded radiographically as anatomical in 80 cases, imperfect in 37 cases, and poor in 10 cases (Table 2), which correlated with the type of fracture classified as elementary or associated-type (chi square = 6.689, *P* = 0.035). Both X and Y showed statistically significant correlation with the quality of fracture reduction (rho = 0.817 and 0.656 respectively, *P* < 0.001 for both). As shown in Figure 2, the mean X and Y were 3.5 mm and 2.6 mm respectively when an imperfect fracture reduction was achieved, which then reached 8.2 mm and 6.1 mm in poorly reduced acetabular fractures (Figure 3).

## Discussion

To recover a functional and pain-free hip is the main goal in the treatment of acetabular fracture. Among the identified poor prognostic factors, unfavorable fracture reduction is considered the most important one leading to biomechanical alteration and accelerated degenerative changes in hip joint[14, 15]. Previously, the restoration of HJC was merely investigated during the postoperative assessments of the quality of reduction in acetabular fractures. In this study, prior to further biomechanical investigation and clinical follow-up studies, we examined the radiographic restoration of HJC following ORIF of acetabular fracture. The results showed a 2.8 mm horizontal shift and a 2.2 mm vertical shift of postoperative HJC in average, which correlated with the radiographically graded quality of fracture reduction.

The biomechanical importance of an anatomically restored HJC has been widely investigated in THA and revision surgeries. Superior or lateral displacement of HJC, causing a decreased moment arm of abductor muscles, was testified to generate increased hip load during gait cycles and lead to higher rate of implant wear and loosening in THA[16–18]. Using mathematical models, Bicanic reported a 0.7% or 0.1% increase of hip load respectively, following every millimeter of lateral or proximal shift of HJC[9]. Similar in the opposite way, the hip load would decrease when the HJC shifted medially or distally. Considering an acetabular fracture, the alteration of the loading pattern was believed to be more complicated[4]. In our study, the majority of the cases presented varying degrees of medial and proximal shifts of HJC. It’s hard, therefore, to clarify the changes of hip load caused by the shifted position of HJC in our study, unless further biomechanical studies could be conducted. However, since the mean values of the HJC shifts appeared to be relatively small when an anatomical or imperfect fracture reduction was achieved (Figure 2), the subsequent changes of hip load would probably be acceptable[19]. Considering poorly reduced fractures, which might bear more clinical significance, to what extent will the shift of HJC affect the hip load changes and the clinical outcomes should be closely analyzed.

Besides the hip load, a shifted HJC may also lead to the changes of surrounding muscle forces in order to balance the moment of body weight. Delp observed a 44% decrease of abduction force and a 27% decrease of flexion force following 2 cm proximal shift of HJC[20]. A 2 cm medial shift of HJC, in the same study, was testified to reduce 26% of the adduction force. In our study, again, the mean values of postoperative HJC shifts were relatively small compared to a 2 cm scale. Therefore the potential contribution of the shifted HJC to the subsequent muscle imbalance and gait changes might be trivial. However, future studies using experimental or computer models would be needed to provide direct evidence for this hypothesis.

Radiographic criteria suggested by Matta are generally used to evaluate the quality of fracture reduction[11]. In our study, an anatomical reduction was achieved in 75.0% of the elementary fractures and in 53.5% of the associated fractures, while the rate of poor reduction was 3.6% and 11.3% respectively. These were comparable with the results of the other studies[2, 21]. An important finding of our study was that the postoperative shifts of HJC were correlated with the quality of fracture reduction. This was reasonable since anatomical reduction would theoretically lead to an ideal restoration of HJC, while a poorly reduced fracture might leave residual displacements of columns and/or walls to hinder the restoration of HJC. Based on this finding, the quality of fracture reduction graded using Matta’ criteria might imply the status of HJC restoration. An anatomical fracture reduction, therefore, should be aimed and checked intraoperatively to restore an optimal HJC.

In this study, the horizontal shift of HJC was found to be correlated with the fracture types. This reflected the clinical reality that an associated-type or so-called complex acetabular fracture would lead to an increased duration of surgery, a decreased quality of fracture reduction, and a higher value of horizontal shift of HJC. Specifically, patients with a both-column or T-shape type of fracture presented highest value of horizontal shift of HJC. Meanwhile, the highest rate of poor functional outcome, as reported by Briffa’s, was observed in the patients with a posterior column, posterior column and posterior wall, or posterior wall type of fracture[2]. This inconsistency between the radiographic and functional evaluations was also reported by Magill previously[22]. As a potential influencing factor for the horizontal shift of HJC, the displacement of the quadrilateral plate was not analyzed in this study because it’s not specifically considered in the Matta’s grading system.

Various methods have been reported to determine the anatomical HJC on two-dimensional pelvic radiographs. Anatomical landmarks like teardrops, Shenton’s line, Köhler’s line, and inter-sacroiliac line were used by different investigators, while the HJC was testified to be most precisely determined referring to the teardrops[23]. However, in our pilot study, the ipsilateral teardrop could only be precisely identified in less than 20% of the postoperative pelvic radiographs due to fracture disruption or implant obstruction. Therefore we used the contralateral intact acetabulum and femoral head as mirrored template to determine the estimated HJC. Similar methods were reported previously in other studies, showing acceptable accuracy and repeatability[9, 24].

This study has a few limitations. First, we only used two-dimensional radiographs to examine the postoperative HJC. Although it’s a common practice in hip arthroplasty, the consistence between an HJC identified in anteroposterior radiographs and that located using functional method was questioned recently[25]. CT scan, three-dimensional image analysis or even computer navigation system, if practical, might provide better information considering HJC location and shape changes of the acetabulum in future studies. Second, we didn’t evaluate biomechanical alterations or follow-up data of functional outcome caused by the shift of HJC in the current study. Based on our findings, special emphasis will be placed on the patients with poor quality of fracture reduction, to investigate the biomechanical consequence as well as functional changes caused by the shift of HJC in our further studies.

## Conclusions

In conclusion, varying degrees of medial and proximal shifts of HJC were observed in the majority of the acetabular fractures following ORIF. The postoperative restoration of HJC showed significant correlation with the quality of fracture reduction. A perfect fracture reduction should be aimed to achieve appropriate HJC restoration. Further studies are required to address the effects of HJC shift on the biomechanical changes and clinical outcomes of hip joint, especially in poorly reduced acetabular fractures.

## Authors’ original submitted files for images

Below are the links to the authors’ original submitted files for images. Authors’ original file for figure 1 Authors’ original file for figure 2 Authors’ original file for figure 3

## Abbreviations

AP: Anteroposterior
CT: Computed tomography
HJC: Hip joint center
ICC: Interclass correlation coefficient
MRD: Maximum residual displacement
ORIF: Open reduction and internal fixation
THA: Total hip arthroplasty
VA line: Vertical axis of the pelvis
X: Horizontal shift of hip joint center
Y: Vertical shift of hip joint center.
