# Supplementary figures and images for: Radiographic analysis of the restoration of hip joint center following open reduction and internal fixation of acetabular fractures: a retrospective cohort study
Source: BMC Musculoskelet Disord. 2014 Aug 13;15:277. doi: 10.1186/1471-2474-15-277 (PMC4137070; doi:10.1186/1471-2474-15-277)

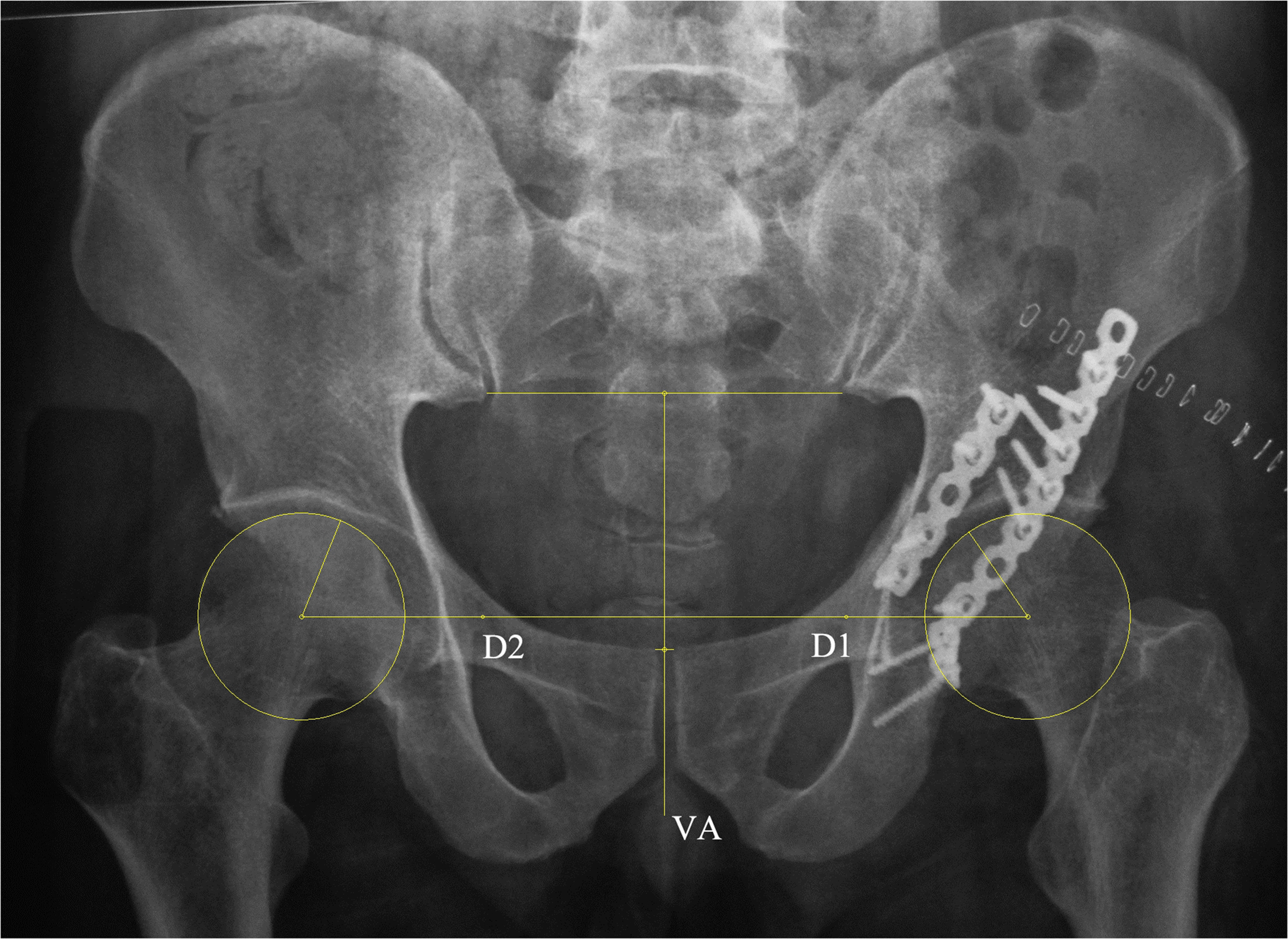

Supplement: Supplementary file 1 — Authors’ original file for figure 1 [file 12891_2013_2218_MOESM1_ESM.tif]

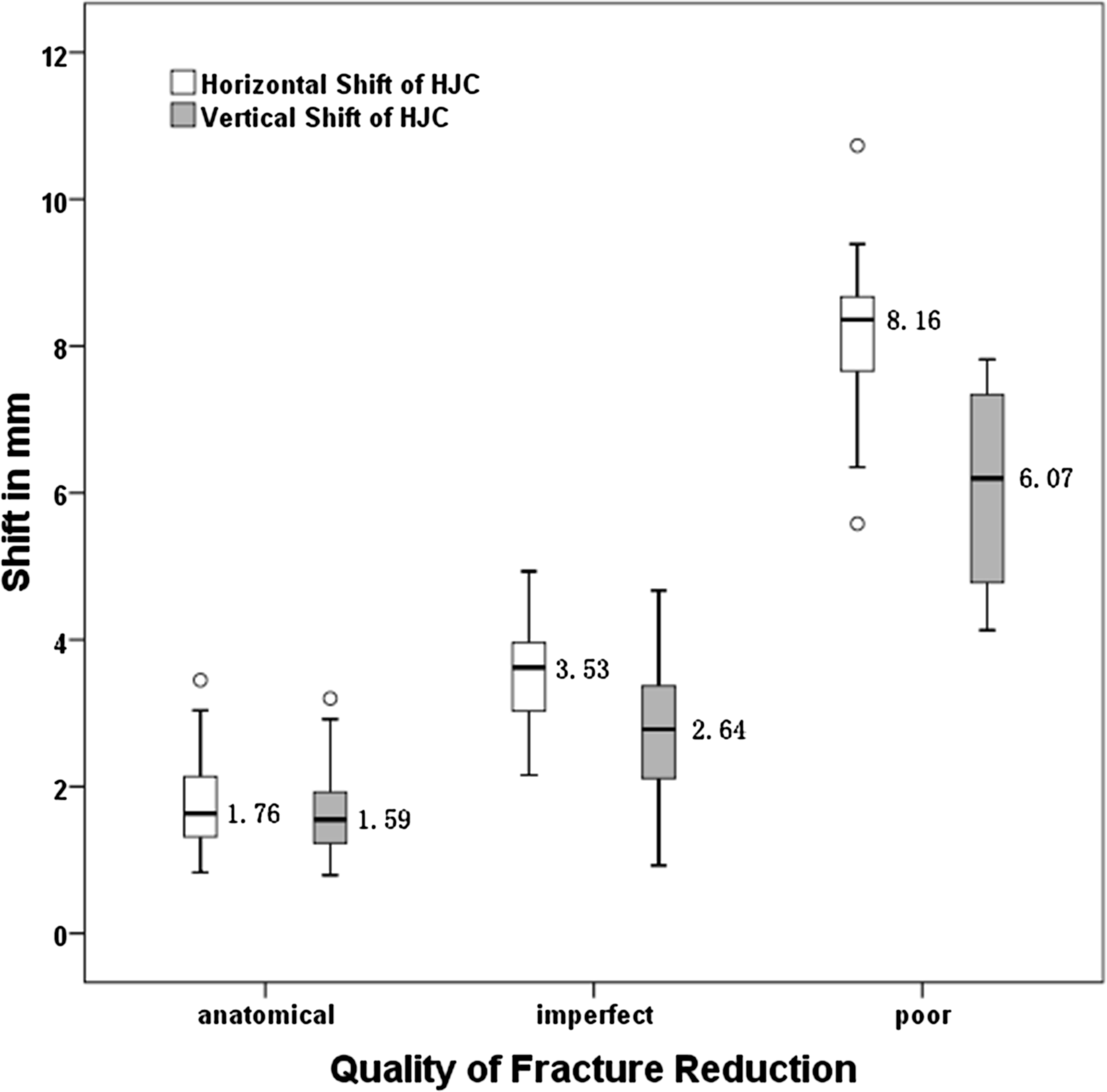

Supplement: Supplementary file 2 — Authors’ original file for figure 2 [file 12891_2013_2218_MOESM2_ESM.tif]

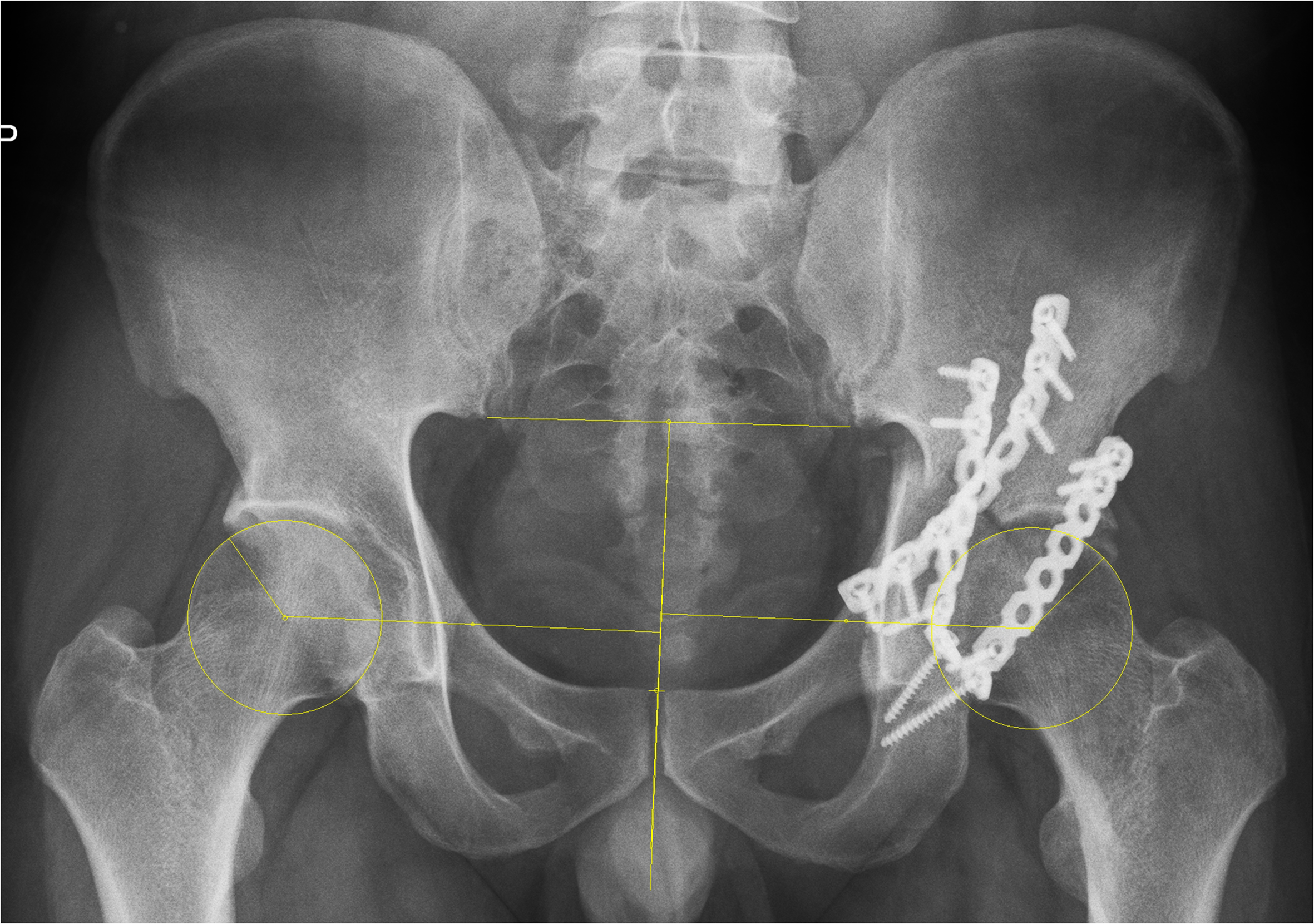

Supplement: Supplementary file 3 — Authors’ original file for figure 3 [file 12891_2013_2218_MOESM3_ESM.tif]
